# Is there a different rating of perceived exertion in men with type 2 diabetes mellitus?

**DOI:** 10.1007/s40200-023-01261-x

**Published:** 2023-08-16

**Authors:** Leon Schwensfeier, Thorsten Kreutz, Christian Brinkmann

**Affiliations:** 1 Institute of Cardiovascular Research and Sport Medicine, Department of Preventive and Rehabilitative Sport Medicine, German Sport University Cologne, Am Sportpark Müngersdorf 6, 50933 Cologne, Germany; 2 Department of Fitness & Health, IST University of Applied Sciences, Düsseldorf, Germany

**Keywords:** Rating of perceived exertion, RPE, Exercise, Sports, Diabetes, T2DM

## Abstract

**Objective:**

Studies show that patients with type 2 diabetes mellitus (T2DM) do not engage in regular exercise as often as individuals without T2DM. In addition to numerous barriers to engaging in regular exercise, a different rating of perceived exertion (RPE) during physical activity has been hypothesized to play a role. Therefore, this study investigates whether T2DM affects RPE.

**Methods:**

RPE values (BORG scale ratings) and heart rate (HR) data were analyzed during an endurance step test (25 W + 25 W every 2 min) at different workloads relative to the individual maximum load (50%, 70% and 90% of peak workload (W_peak_)) in patients with T2DM and in non-diabetic control (CON) subjects (n = 12 in each group). Furthermore, in a larger group of overweight patients with T2DM (n = 81), it was investigated whether glycated hemoglobin (HbA1c) levels correlate with RPE values at the different relative loads.

**Results:**

Neither RPE nor HR values significantly differed between T2DM and CON subjects at 50%, 70% or 90% of W_peak_. No significant correlations were identified between HbA1c levels and RPE values.

**Conclusion:**

There is no evidence in our study that T2DM leads to a different perception of physical exertion. Other causes must therefore be responsible for the increased lack of motivation of T2DM patients to engage in regular exercise.

## Introduction

According to the International Diabetes Federation’s (IDF) Diabetes Atlas [1], type 2 diabetes mellitus (T2DM) is the most common type of diabetes, accounting for over 90% of all diabetes cases worldwide. Regular physical activity is considered an essential cornerstone in the prevention and treatment of T2DM [2, 3]. Nevertheless, studies show that individuals with T2DM are more inactive than individuals without T2DM and do not engage in regular exercise as often as non-diabetic individuals [4–9].

In addition to numerous barriers to engaging in regular exercise (e.g., immobility, lack of time, lack of social support), a different subjective perception of physical exertion has been deemed to be a barrier [10–12]. A relationship generally exists between the subjective perception of the intensity of physical exertion and levels of physical activity [13–15].

To date, it is unclear whether there are differences in the rating of physical exertion between men with and without T2DM, whereas some data for women with and without T2DM are already available [16, 17].

This study investigates whether men with T2DM perceive exertion to be more strenuous than men of similar age, weight and peak workload without T2DM. Ratings of perceived exertion (RPE) and heart rate (HR) values will be analyzed during an endurance step test at different workloads relative to the individual maximum load (50%, 70% and 90% of peak workload (W_peak_)). In addition, whether glycated hemoglobin (HbA1c) levels correlate with the ratings of the intensity of the different physical loads will be investigated among a larger group of overweight men with T2DM.

## Methods

### Study design

This study uses pooled data from studies of overweight people with and without T2DM. All studies took place between 2007 and 2019 at the Institute of Cardiovascular Research and Sport Medicine (Department I. Preventive and Rehabilitative Sport Medicine), German Sport University Cologne. All studies were approved by the Ethics Committee of the German Sport University Cologne and were in line with the Declaration of Helsinki.

### Subjects

The inclusion criteria for patients with T2DM were defined as follows: medical diagnosis for T2DM, male sex, 40–80 years of age, overweight (body mass index (BMI) ≥ 25 kg/m^2^), no treatment with insulin. For individuals without T2DM, the inclusion criteria were as follows: male sex, overweight (BMI ≥ 25 kg/m^2^) and fasting glucose < 126 mg/dl or HbA1c < 6.5%. All persons should be untrained (i.e., engaging in exercise no more than once a week).

The general exclusion criteria for all subjects were severe diseases other than factors of metabolic syndrome (hypertension, hyperlipidemia).

For the first part of the study (direct comparison of RPE and HR values between T2DM patients and non-diabetic subjects), 12 T2DM patients were selected from a larger patient pool by applying propensity score matching [18]. They were matched as closely as possible with 12 non-diabetic subjects in terms of age, BMI, and peak workload during exercise testing. Subjects’ characteristics are listed in Table 1. Some of the patients were taking pharmaceutical drugs. These are listed in Table 2.

**Table 1:** Anthropometric data and health variables of subjects with and without type 2 diabetes mellitus (T2DM), n=24

	Subjects with T2DM (n = 12)	Non-diabetic subjects (n = 12)	p value
Age [years]	57.1 ± 7.1	55.1 ± 6.6	0.862^#^
BMI [kg/m^2^]	31.0 ± 4.9	31.5 ± 3.1	0.744^*^
W_peak_ [W]	171 ± 40	167 ± 27	0.766^*^
HR_peak_ [bpm]	151 ± 22	146 ± 19	0.532^*^
Fasting glucose [mg/dl]	143 ± 36	102 ± 13 (n = 11)	0.002^*^
HbA1c [%]	6.8 ± 1.0	5.6 ± 0.3 (n = 5)	0.002^#^
Duration of T2DM [years]	4.8 ± 2.6	/	/

BMI: body mass index; W_peak_: peak workload; HR_peak_: peak heart rate; HbA1c: glycated hemoglobin; * t-test; # Mann-Whitney U test

**Table 2:** Subjects’ medication I

	Anti-diabetic/ blood glucose lowering drugs	Anti-hypertension drugs	Lipid-lowering drugs	Other drugs (e.g., acetylsalicylic acid, L-thyroxine, pantoprazole)
Subjects with T2DM, n = 12	n = 11	n = 7	n = 2	n = 7
Non-diabetic subjects, n = 12	n = 0	n = 5	n = 2	n = 7

T2DM: type 2 diabetes mellitus

A patient pool of 81 men with T2DM was used for the planned simple correlation analyses between HbA1c and RPE values at 50%, 70% and 90% of individual W_peak_. The subjects’ characteristics are presented in Table 3, and the participants’ pharmaceutical drugs are shown in Table 4.

**Table 3:** Anthropometric data and health variables of subjects with type 2 diabetes mellitus (T2DM), n = 81

Age [years]	62.5 ± 6.9
BMI [kg/m^2^]	33.1 ± 5.3
W_peak_ [W]	136 ± 34
Fasting glucose [mg/dl]	161 ± 47
HbA1c [%]	7.1 ± 1.2
Duration of T2DM [years]	6.8 ± 6.8

BMI: body mass index, W_peak_: peak workload, HbA1c: glycated hemoglobin

**Table 4:** Subjects’ medication II

	Anti-diabetic/ blood glucose lowering drugs	Anti-hypertension drugs	Lipid-lowering drugs	Other drugs (e.g., acetylsalicylic acid, L-thyroxine, pantoprazole)
Subjects with T2DM, n = 81	n = 69	n = 60	n = 23	n = 44

T2DM: type 2 diabetes mellitus

### Cycling ergometry and rating of perceived exertion

The cycling ergometry was performed in accordance with the World Health Organization’s (WHO) load scheme described in [19]. The starting load of 25 W was increased by 25 W every 2 min. W_peak_ was defined as the last load level of cycling ergometry that the subjects continued to perform for at least 1:45 mins. At the end of each load level (15 s before the end), the subjects’ RPE was checked using the BORG scale [20]. It represents a valid instrument for estimating the intensity of perceived physical activity in healthy people and in those with chronic diseases [21]. The subjects’ HR was recorded by an electrocardiogram throughout the cycling ergometry. Subjects were tested using the following dropout criteria:

muscular exhaustion, angina pectoris, ischemia, cyanosis, respiratory insufficiency, frequent arrhythmia, blood pressure > 250/115 mmHg, and other complaints (dizziness, paleness, coordination problems).

The selection of the three exercise intensities (50%, 70% and 90% of individual W_peak_) is based on exercise intensity levels used in the literature [22–24]. Accordingly, 50% of the maximum power is considered moderate, 70% moderate-intense, and 90% highly intense exercise.

### Statistical analyses

Statistical analyses were performed using the IBM SPSS Statistics 29 program (IBM, Chicago, IL, USA). In addition, XLSTAT software version 9.0 (Addinsoft, Paris, France) was used to perform propensity score matching [18] in the first part of the study. The matching followed defined criteria and allowed comparability of the subjects based on the variables age, BMI and W_peak_. After testing for normal distribution using the Shapiro-Wilk test, the data of subjects with T2DM (n = 12) were compared with those of the subjects without T2DM (n = 12) using either the unpaired t-test for normally distributed data or the Mann-Whitney U test for non-normally distributed data. In the second part of the study (subjects with T2DM, n = 81), Spearman correlation analyses were performed to analyze possible relationships between HbA1c levels (non-normally distributed data) and RPE values.

The significance level was set at p < 0.05. Values are means ± standard deviation (SD). Raw data were initially prepared using Microsoft Excel 16. RPE and HR values were calculated by linear interpolation in Excel when necessary. RPE values that were given as a range (e.g., 14–15) were replaced with the mean (e.g., 14.5). Missing values were replaced by the mean of the last and next value. If one of these values was not available, no replacement was made.

## Results

### Comparison of ratings of perceived exertion between type 2 diabetes mellitus patients and non-diabetic subjects

**Fig. 1:**
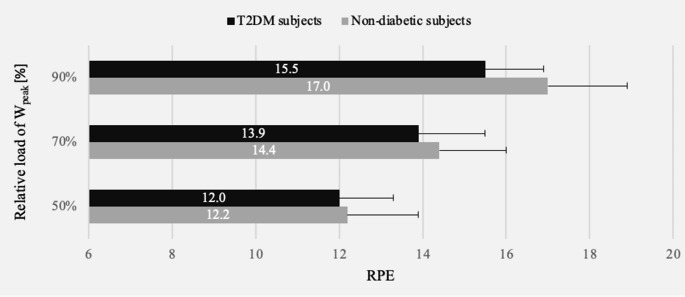
Rating of perceived exertion (RPE) values of subjects with type 2 diabetes mellitus (T2DM) compared to those of non-diabetic subjects. W_peak_: peak workload. 50% of W_peak_: n = 24, 12 vs. 12, t-test: p = 0.740; 70% of W_peak_: n = 23, 12 vs. 11, U test: p = 0.388; 90% of W_peak_: n = 17, 9 vs. 8, t-test: p = 0.093

Fig. 1 illustrates the subjective perception of exertion in patients with T2DM and non-diabetic subjects at three relative exertion intensities. The RPE values were not significantly different between T2DM and non-diabetic individuals at 50%, 70% or 90% of W_peak_.

Table 5 presents the HR values of patients with T2DM and non-diabetic subjects at the three different levels of exertion. There were no significant differences in HR values between T2DM and non-diabetic individuals at all three loads (50%, 70%, 90% of W_peak_).

**Table 5:** Absolute and relative heart rate (HR) values of subjects with and without type 2 diabetes mellitus (T2DM)

	T2DM subjects	Non-diabetic subjects	p value
Absolute HR at 50% of W_peak_ [bpm]	117 ± 16	110 ± 9	0.178^*^
Relative HR at 50% of W_peak_ [% of peak heart rate]	77.6% ± 5.0%	75.5% ± 6.0%	0.344^*^
Absolute HR at 70% of W_peak_ [bpm]	132 ± 18	124 ± 13	0.246^*^
Relative HR at 70% of W_peak_ [% of peak heart rate]	87.3% ± 4.3%	84.2% ± 3.8%	0.083^*^
HR at 90% of W_peak_ [bpm]	147 ± 22	138 ± 18	0.374^*^
Relative HR at 90% of W_peak_ [% of peak heart rate]	96.1% ± 1.3%	95.0% ± 1.6%	0.091^#^

HR: heart rate; W_peak_: peak workload. 50% W_peak_: n = 24 (12 vs. 12); 70% W_peak_: n = 23 (12 vs. 11); 90% W_peak_: n = 17 (9 vs. 8). * t-test, # Mann-Whitney U test

### Correlation analyses between glycemic control and ratings of perceived exertion

**Fig. 2:**
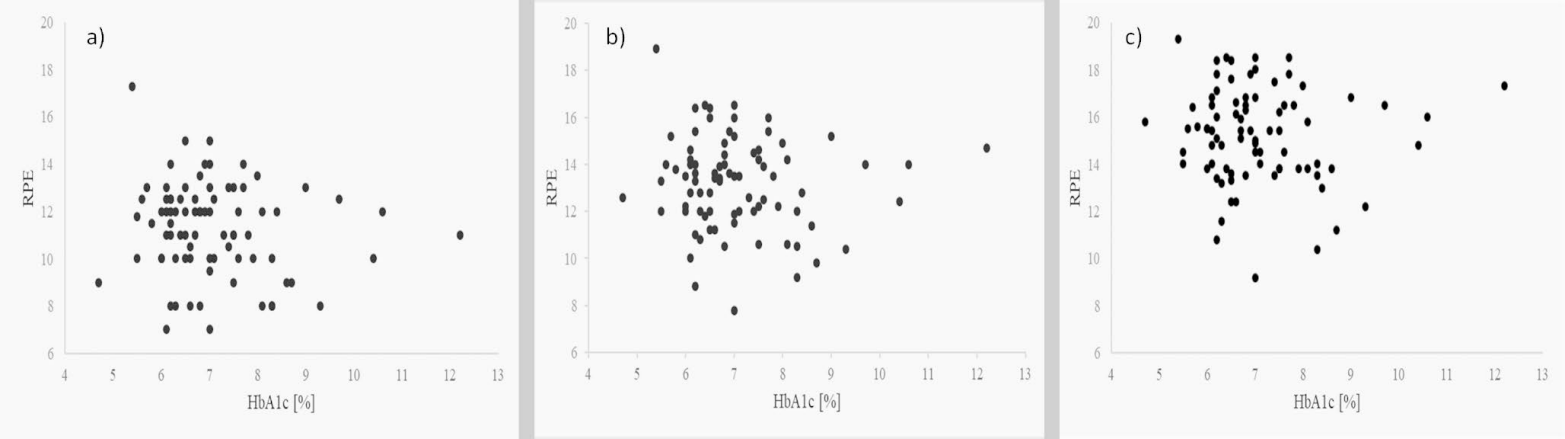
Correlation analyses between glycated hemoglobin. (HbA1c) and the rating of perceived exertion (RPE) values at **a**) 50%, **b**) 70% or **c**) 90% of peak workload (W_peak_) in subjects with type 2 diabetes mellitus (T2DM). n = 81. Spearman correlation analyses: no significant results (50% of W_peak_: Spearman’s rho=-0.110, p = 0.329; 70% of W_peak_: Spearman’s rho=-0.082, p = 0.465; 90% of W_peak_: Spearman’s rho=-0.086, p = 0.445)

There were no significant correlations between HbA1c and RPE values for any of the three relative exercise intensities in the larger group of T2DM patients. The data points are shown in Fig. 2.

## Discussion

The present results do not provide support for a difference in the subjective perception of exertion in men with T2DM and the influence of glycemic control on the rating of an exertion’s intensity. At none of the three exercise intensities (at 50%, 70%, and 90% of W_peak_) during cycling ergometry, significantly different RPE values were observed in men with T2DM compared with those of men with a similar age, BMI and W_peak_ without T2DM.

RPE values at the different loads were similar between T2DM and non-diabetic individuals at similar absolute and relative heart rates. Therefore, a possible effect of T2DM on RPE could have been more easily recognized in this study, as it is generally accepted that the HR can affect RPE [25].

In addition, there were no significant correlations between HbA1c values and the ratings of perceived exertion in the larger group of overweight T2DM patients (n = 81).

To date, only studies on the perception of exertion among women with T2DM are available. In this regard, Huebschmann et al. [16] showed that at low intensities (20 and 30 W), RPE values were significantly higher in women with T2DM (n = 13) than in normal weight or overweight women without T2DM (both n = 13). However, after adjustment for fitness level, the finding only applied for 20 W, which is a relatively low load (exercise loads are usually higher). In a follow-up study [17], women with and without T2DM (n = 26, n = 28) were included. There were no significant differences in the perception of exertion at 30 W and at 35% of peak oxygen uptake (VO_2peak_).

However, there are theoretical considerations that patients with T2DM could have an increased sense of exertion. It has been demonstrated that ratings of perceived exertion are also closely correlated with lactate values and that T2DM patients often have hyperlactatemia [25, 26]. Lactate was not analyzed in this study. In future studies, analyses of lactate values could provide additional information.

One of the limitations of the present study is that some of the subjects were taking medication that may have had an influence on the ratings of perceived exertion. For example, it has been reported that taking beta-blockers can alter the assessment of exercise intensity by RPE values [27]. Some studies have demonstrated an effect of metformin on subjective RPE [26, 28], while others have not [29–31]. The evidence is not entirely clear.

Furthermore, RPE is presumably also influenced by training experience. People with a lot of training experience know their load limit better. Therefore, RPE may also be subject to a learning effect [32]. However, all subjects in the study were untrained and had relatively low training experience (i.e. exercise no more than once a week). In addition, the relatively small number of participants for comparing men with and without T2DM is a clear limitation of the study. This is attributable to the fact that there were only 19 non-diabetic control subjects in our subject pool and that propensity score matching could not assign closely matched persons to all of them. Larger studies are needed to confirm the present result. Furthermore, HbA1c values differed significantly between groups, but the means were not very far apart. It is possible that differences in RPE values only become apparent when differences in HbA1c values are larger.

A particular strength of this study is the selection of higher intensities compared to those in previous studies [16, 17]. The selected loads correspond to realistic exercise loads in the moderate-intense or highly intense range.

## Conclusion

In our study, there is no evidence that T2DM leads to a different sense of exertion. Other causes must be responsible for the increased inactivity observed in many T2DM patients and their demotivation to engage in regular exercise.

Since a higher perception of exertion is obviously not a barrier to physical activity in patients with T2DM, physicians should take the opportunity to design exercise programs tailored to individual needs and preferences. Reasons that discourage T2DM patients from regular exercise (e.g. lack of enjoyment, lack of social support, lack of confidence in their own abilities, etc.) should be assessed in one-on-one interviews, and strategies should be developed to address these barriers [33].
